# Functional Deficits in *Pak5*, *Pak6* and *Pak5/Pak6* Knockout Mice

**DOI:** 10.1371/journal.pone.0061321

**Published:** 2013-04-08

**Authors:** Melody A. Furnari, Michelle L. Jobes, Tanya Nekrasova, Audrey Minden, George C. Wagner

**Affiliations:** 1 Joint Program in Toxicology, Rutgers, The State University of New Jersey, Piscataway, New Jersey, United States of America; 2 Susan Lehman Cullman Laboratory for Cancer Research, Department of Chemical Biology, Ernesto Mario School of Pharmacy, Rutgers, The State University of New Jersey, Piscataway, New Jersey, United States of America; 3 Department of Psychology, Rutgers, The State University of New Jersey, Piscataway, New Jersey, United States of America; Alexander Flemming Biomedical Sciences Research Center, Greece

## Abstract

The p21-activated kinases are effector proteins for Rho-family GTPases. PAK4, PAK5, and PAK6 are the group II PAKs associated with neurite outgrowth, filopodia formation, and cell survival. *Pak4* knockout mice are embryonic lethal, while *Pak5, Pak6*, and *Pak5/Pak6* double knockout mice are viable and fertile. Our previous work found that the double knockout mice exhibit locomotor changes and learning and memory deficits. We also found some differences with *Pak5* and *Pak6* single knockout mice and the present work further explores the potential differences of the *Pak5* knockout and *Pak6* knockout mice in comparison with wild type mice. The *Pak6* knockout mice were found to weigh significantly more than the other genotypes. The double knockout mice were found to be less active than the other genotypes. The *Pak5* knockout mice and the double knockout mice performed worse on the rotorod test. All the knockout genotypes were found to be less aggressive in the resident intruder paradigm. The double knockout mice were, once again, found to perform worse in the active avoidance assay. These results indicate, that although some behavioral differences are seen in the *Pak5* and *Pak6* single knockout mice, the double knockout mice exhibit the greatest changes in locomotion and learning and memory.

## Introduction

The p21-activated kinases (PAKs) are effector proteins for the Rho-family GTPases Cdc42 and Rac. Cdc42 and Rac are molecular switches involved in several cell processes including cell adhesion and migration as well as apoptosis [1]. Cdc42 and Rac exist in two forms, GDP-bound and the activated GTP-bound form in which they can interact with the PAKs and cause downstream effects in the cell [1]. There are two families of PAK in mammals; group I which includes PAK1, PAK2, and PAK3, and group II which includes PAK4, PAK5, and PAK6. These families are based on structure and homology. Group I PAKs have very similar sequences and are distinguished by an amino-terminal regulatory domain and a carboxyl-terminal kinase domain [2], [3], [4]. The regulatory domain contains a Cdc42/Rac-interactive binding (CRIB) domain that mediates PAK binding to Cdc42 and Rac. The group II PAKs, however, are more dissimilar with varying proline-rich potential SH3-domain-binding sites and, except for PAK5, do not contain the auto-inhibitory domain [5]. The group II PAKs bind preferentially to Cdc42. However, their kinase activity can occur independent of Cdc42 binding [6].

There are also differences in expression of the group I and group II PAKs. In the group I PAKs, PAK1 is expressed mostly in the brain, but also in the muscles and spleen. PAK2 is expressed ubiquitously while PAK3 is expressed exclusively in the brain [2], [3], [7], [8]. For the group II PAKs the highest expression of PAK4 is in the prostate, testes, and colon, although it is ubiquitously expressed; PAK5 is most highly expressed in the brain but also expressed in the adrenal gland, pancreas, and other tissues; and PAK6, while also having high expression levels in the brain, is expressed in the prostate, testes, thyroid, and placenta among other tissues [9], [10], [11], [12].

PAK4 and PAK5 have been associated with neurite outgrowth and filopodia formation, while PAK6 has a role in the regulation of the activity of the androgen receptor. PAK6 activity has been shown to be elevated when interacting with the androgen receptor in a pathway independent of Rho GTPases [9], [13]. PAK6 lowers the level of transcriptional activity of both the androgen receptor and estrogen receptor [9], [13]. PAK4 and PAK5 have been shown to regulate neurite outgrowth. PAK4 deactivates cofilin by activating LIMK (LIM domain kinase 1) which then inhibits neurite outgrowth [14]. PAK5, when overexpressed, causes an increase in filipodia formation and induces neurite outgrowth [15]. PAK4 and PAK5 also activate the JNK (c-Jun N-terminal kinase) pathway which is involved in apoptosis and cell survival in response to stress [11], [16]. Knocking out these genes in mice has lead to deficits in cortical neurons such as abnormal growth cones, fewer neurite outgrowths, and abnormal filopodia formation. These types of changes often result from the changes in cytoskeletal dynamics. Proper morphology and arrangement of synaptic contacts are considered essential for learning and memory and the changes and defects in these knockout mice provide evidence of a link between the group II PAKs and learning and memory.

*Pak* knockout mice have been developed to further investigate the biological functions of the group II PAKS. *Pak4* knockout is embryonic lethal in mice, while *Pak5*, *Pak6* and *Pak5/Pak6* double knockout mice are viable and fertile [12], [17], [18]. Based on our preliminary work, *Pak5/Pak6* double knockout mice exhibit locomotor changes as well as subtle learning and memory deficits compared to wild type controls [18]. *Pak4* has been associated with motor neuron development [17]. Because *Pak4, Pak5,* and *Pak6* are all members of the group II Pak family behavioral tests involving motor neurons such as open field activity and the rotorod were used to evaluate the motor neuron function in mice lacking these genes. In addition to PAK4’s involvement in motor neuron development, PAK5 and PAK6 are expressed in the brain and specifically PAK4 *and* PAK5 has been shown to be involved in regulation of neurite outgrowth [9], [10], [11], [12], [14], [15]. Therefore, a learning and memory behavioral test, the active avoidance t-maze was also utilized to determine if the knockout mice had any cognitive impairment. The previous work with double knockout mice indicated that the double knockout mice had decreased levels of aggression and increased weight [18]. Given that PAK6 inhibits androgen receptor signaling, and the androgen receptor is involved in weight determination and aggression, weight was monitored and the resident intruder aggression test was used to determine if PAK6 was responsible for the increased weight and decreased aggression seen in the double knockout mice[9], [13], [19]. In the present study, *Pak5* and *Pak6* single knockout mice were assessed using the same battery of behavioral tests as in the previous study to determine if one gene or the other contributed to the deficits seen in the double knockout mice. A portion of the data presented in this report was included as preliminary and supplementary observations reported in our prior publication [18]. The supplementary data included an activity assay, active avoidance, elevated plus maze, and the resident intruder paradigm, although these were not fully analyzed and only included a portion of the data presented in the present work. The data are now embedded in the present report and have now been expanded and fully analyzed.

## Materials and Methods

### Ethics Statement

All procedures were conducted in strict compliance with the policies on animal welfare of the National Institute of Health. The protocol was approved by the Animal Care and Use Committee at Rutgers University (87-060).

### Animals

*Pak5* knockout [12], *Pak6* knockout and *Pak5*/*Pak6* double knockout (DKO) mice [18] were bred in the Laboratory for Cancer Research, Rutgers University. *Pak5* and *Pak6* knockout mice were backcrossed to C57BL/6J strain nine times and contained 99.8% of C57BL/6J genes. DKO and wild type mice were generated from the intermediate cross and both contained 85% of C57BL/6J background genes. In addition, *Pak5* knockout mice (stock 015827), *Pak6* knockout mice (stock 015826), and *Pak5/Pak6* double knockout mice (stock 015825) can be obtained from Jackson Laboratories (Jackson Laboratories; Bar Harbor, ME). Five male double knockout mice, five male *Pak5* knockout mice, eight male *Pak6* knockout and eight male wild type mice were used in these studies. Mice were housed in individual hanging wire cages (20 cm×10 cm×12 cm) with free access to food and water in a temperature and humidity regulated room with a 12 hr light/dark cycle. Body weights were measured weekly. All of the experiments were conducted with the experimenter blind to mouse genotypes.

### Open Field Activity

The open field activity test evaluates the baseline activity level of mice in an open space. Mice were taken out of their home cages and placed in activity chambers consisting of a Plexiglas box (42×22×14 cm) with six photocell sensors placed 7 cm apart and 2.5 cm above the floor of the chamber. The number of times the mouse broke the photocell beams was recorded every five minutes. This is counted every five minutes because normally mice are hyperactive and explore when they are first placed in the chamber. Then as time goes on, the number of beam breaks plateaus and differences in activity can be evaluated.

### Elevated Plus Maze

The elevated plus maze evaluates anxiety. Normally, mice will not cross into the open arm because elevated open spaces make them anxious. In addition, an increase in fecal boli can also indicate a higher level of anxiety. The elevated plus maze was a “plus” shaped apparatus 60 inches above the floor with two long closed arms (65 cm long and 8 cm wide), two short open arms (30 cm long and 9 cm wide), and a central neutral 5 cm by 5 cm square. Each mouse was placed in the center square and observed for 10 minutes. The number of times the animal crossed into a closed arm, open arm, or jumped off the maze was recorded as was the number of fecal boli.

### Rotorod

The rotorod test evaluates motor coordination and balance. A shortened latency for the mouse to fall from the rod indicates a deficit in motor coordination and balance. Each mouse was placed on the rotorod with a diameter of 6 cm, rotating at 12 revolutions per minute. The rotorod was 60 cm above a padded receptacle. The latency to fall from the rotorod was recorded for each mouse for three trials, with each trial lasting no more than 60 seconds.

### Social Chamber

The social chamber was a 40 cm×40 cm×36.6 cm Plexiglas chamber with a stainless steel grid floor. Within the chamber were two cylinders 11 cm in diameter and 13 cm tall made of the same stainless steel grid as the floor, located in opposite corners of the chamber. Each mouse was given a 10 minute habituation period to explore the chamber before the start of the trial. After 10 minutes an adult male BALB/c mouse was placed in one of the cylinders and the subject mouse was placed back in the center of the chamber. Each time the subject placed one or both paws on a cylinder a contact was recorded. The number of contacts with either the target cylinder containing the BALB/c mouse or the control cylinder containing nothing was recorded. Each mouse was observed for three 10 minute trials over three days. More contact with the target cylinder containing a novel mouse compared to the empty cylinder, indicated a high level of social behavior.

### Aggression

The mice were observed in a resident/intruder paradigm where the *Pak* knockout mice and their wild type controls were “resident” mice individually housed in pan cages with wood chip bedding for two weeks before the assay began. Male C57BL/6 mice that were group housed with up to five mice per cage for at least two weeks before the experiment were used as the intruders. One intruder mouse was placed in the home cage of the resident mouse for one 30 minute trial per day for three days. During the thirty minute trial the latency to the first attack and number of attacks thereafter were recorded as well as which mouse initiated each attack. Mice normally protect their territory by attacking an intruder mouse. Less number of attacks or a slower latency to the first attack indicate less aggression.

### Active Avoidance

Active avoidance was assessed in a T-maze, which had two 20×11 cm areas connected on either side of a 40×10 cm corridor. The Plexiglas walls were 18 cm high and the floor consisted of stainless steel bars 0.75 cm apart wired to a shock generator, except in the “safe” area of the maze (one of the two 20×11 cm areas). Mice were placed at the end of the corridor in the designated start box where the mice were confined for 20 seconds before each trial was started. After 20 seconds, a conditioned stimulus tone was played and door to the start box was opened so the mice could move around the maze. An avoidance was recorded if the mouse reached the “safe” area before 10 seconds had elapsed. If the mouse did not reach the “safe” area within 10 seconds, a 0.8 mA foot shock was sent through the floor of the maze that lasted for a maximum of 10 seconds. If the mouse was able to reach the “safe” area after the shock began, but before 10 seconds was over, it was recorded as an escape response. If the mouse never reached the “safe” area over the course of the 20 second trial, then a response failure was recorded. Each mouse was assessed for 10 trials each day for 5 days. The type of response and the latency to make the response was recorded for each trial. Mice were tested on two separate occasions (an acquisition phase and a retention phase) two months apart. Typically mice learn to avoid the shock and run to the “safe” area during the course of testing. A low number of successful avoidances indicates deficits in learning and memory.

## Results

### *Pak6* knockout mice weigh more than other genotypes

**Figure 1:**
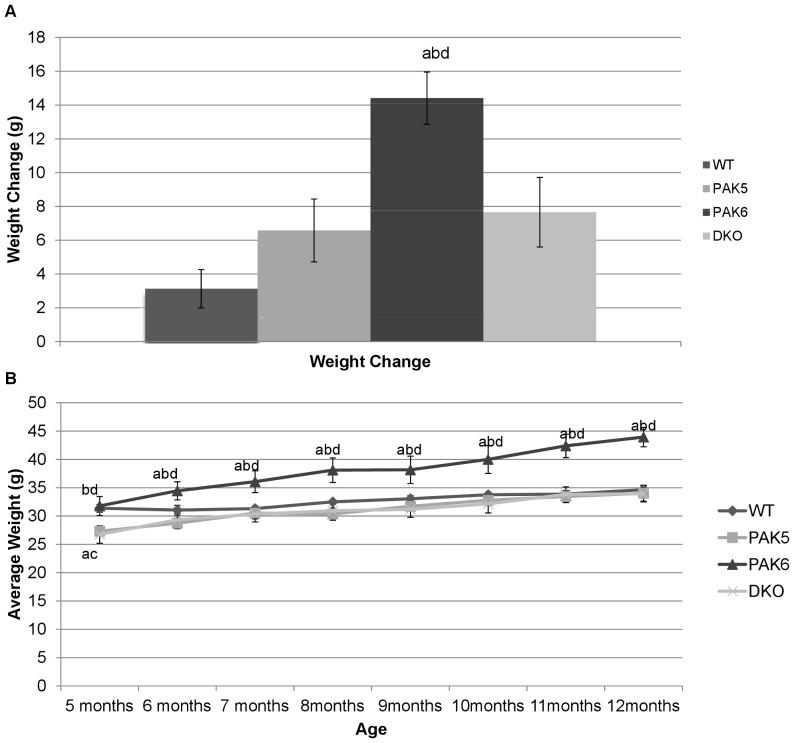
**a. Total Body Weight Change**. PAK6 knockout mice had the largest increase in weight, significantly different from WT (a  =  p<.05 compared to WT), PAK5 (b =  p<.05 compared to PAK5), and DKO (d =  p<.05 compared to DKO). WT n = 8. PAK5 n = 5. PAK6 n = 8. DKO n = 5. **b. Body weight.** PAK6 knockout mice were significantly heavier than PAK5(b) and DKO(d) by 5 months of age and significantly heavier than WT(a) from 6 months of age. The DKO and PAK5 knockout mice weighed significantly less than the WT (a) and PAK6(c =  p<.05 compared to PAK6) at 5 months of age, but weighed similarly to the WT after 6 months of age. WT n = 8. PAK5 n = 5. PAK6 n = 8. DKO n = 5.

An ANOVA was used to analyze the total weight change per genotype from 19 weeks of age to 48 weeks of age. A significant difference in weight change between genotypes was found [F(3,20) = 10.014, p = 0.0003]. *Post hoc* tests revealed the *Pak6* knockout mice gained significantly more weight compared to wild type mice (p<0.0001), *Pak5* knockout mice (p = 0.0028), and double knockout mice (p = 0.0082)[Figure 1a].

A repeated measures ANOVA was run to analyze the average weight per genotype from 5 months of age to 12 months of age. A significant effect of month [F(7, 140) = 55.718, p<0.0001] was found, with weight showing a significant upward trend. A significant effect of genotype [F(3, 140) = 7.341, p = 0.0017] and a significant interaction of month by genotype were also found [F(21, 140) = 3.956, p<0.0001]. *Post hoc* tests showed at 5 months of age the *Pak6* knockout mice were significantly heavier than the *Pak5* knockout mice (p = 0.0154) and the double knockout mice (p = 0.0071), and that the wild type mice were significantly heavier than the *Pak5* knockout mice (p = 0.0263) and the double knockout mice (p = 0.0124). At all other time points the *Pak6* mice were significantly heavier than the other genotypes and the other genotypes weighed similarly [Figure 1b].

### Double knockout mice are less active than other genotypes

**Figure 2:**
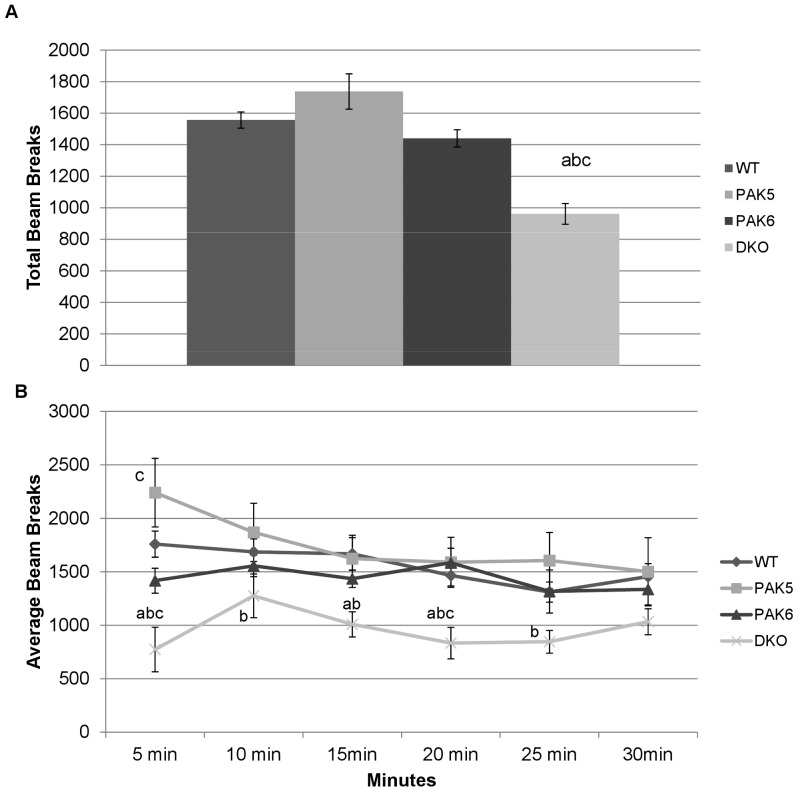
**a. Total activity first run**. The DKO mice were significantly less active than the WT(a), PAK5(b) and PAK6(c) mice. WT n = 8. PAK5 n = 5. PAK6 n = 8. DKO n = 5. **b. Activity over 30 minutes.** The double knockout mice were significantly less active than the wild type mice (a), the PAK5 knockout mice (b), and the PAK6 knockout mice (c). WT n = 8. PAK5 n = 5. PAK6 n = 8. DKO n = 5.

The open field activity assay was done on three separate occasions. On each occasion the mouse was placed in the activity chamber and number of infrared beam breaks was recorded in 5 minute bins over 30 minutes. A repeated measures ANOVA was used to analyze each day. For the first run of the open field activity assay, a repeated measures ANOVA revealed a significant effect of genotype [F(3,110) = 5.256, p = 0.0069]. For average total beam breaks, *post hoc* tests revealed the double knockout mice were significantly less active compared to the *Pak5* knockout mice (p = 0.0011), the *Pak6* knockout mice (p = 0.0178), and the wild type mice (p = 0.0043). The *Pak5* knockout mice, *Pak6* knockout mice, and wild type mice were not found to be significantly different from one another [Figure 2a].

The repeated measures ANOVA also revealed a significant effect of bin [F(5,110) = 4.986, p = 0.0004], with activity showing a significant downward trend over the thirty minute testing period and a significant interaction of genotype by bin [F(15, 110) = 2.047, p = 0.0179]. *Post hoc* tests revealed for the first 5 minute bin that the double knockout mice were significantly less active compared to the *Pak5* knockout mice (p<0.0001), the *Pak6* knockout mice (p = 0.210) and the wild type mice (p = 0.001). During the first 5 minute bin the *Pak5* knockout mice were significantly more active than the *Pak6* knockout mice (p = 0.0043). During the first 5minute bin the *Pak5* knockout mice and the *Pak6* knockout mice were not found to be significantly different from the wild type mice. *Post hoc* tests for the second 5 minute bin revealed that the double knockout mice were significantly less active compared to the *Pak5* knockout mice (p = 0.0325). *Post hoc* tests for the third 5 minute bin revealed that the double knockout mice were significantly less active than the *Pak5* knockout mice (p = 0.140) and the wild type mice (p = 0.0074). *Post hoc* tests for the fourth 5 minute bin showed that the double knockout mice were significantly less active compared to the *Pak5* knockout mice (p = 0.0043), the *Pak6* knockout mice (p = 0.002) and the wild type mice (p = .0074). *Post hoc* tests for the fourth 5 minute bin showed the double knockout mice to be significantly less active compared to the *Pak5* knockout mice (p = 0.0135). *Post hoc* tests for the last 5 minute bin showed no significant differences in activity level of any of the genotypes [Figure 2b].

**Figure 3:**
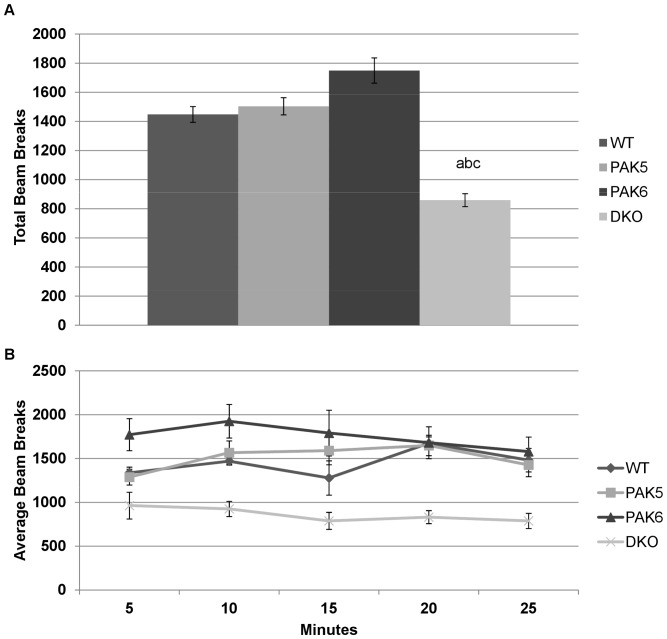
**a. Total Activity second run**. The DKO mice were significantly less active compared to the wild type mice, the PAK5 knockout mice, and the PAK6 knockout mice. WT n = 8. PAK5 n = 5. PAK6 n = 8. DKO n = 5. **b. Second run-Activity over 30 minutes.** There was no difference in activity in each genotype over the thirty minute trial. WT n = 8. PAK5 n = 5. PAK6 n = 8. DKO n = 5.

A repeated measures ANOVA was used to analyze the data from the second run of the open field activity assay. A significant effect of genotype [F(3, 84) = 7.344, p = 0.0015] was found. *Post hoc* tests revealed the double knockout mice had significantly less total beam breaks compared to *Pak5* knockout mice (p = 0.0062), *Pak6* knockout mice (p = 0.0001), and wild type mice (p = 0.0068). The wild type mice, *Pak5* knockout mice, and *Pak6* knockout mice were not found to be significantly different from each other [Figure 3a]. There was no significant difference in activity over time and no interaction of activity over time and genotype [Figure 3b].

**Figure 4:**
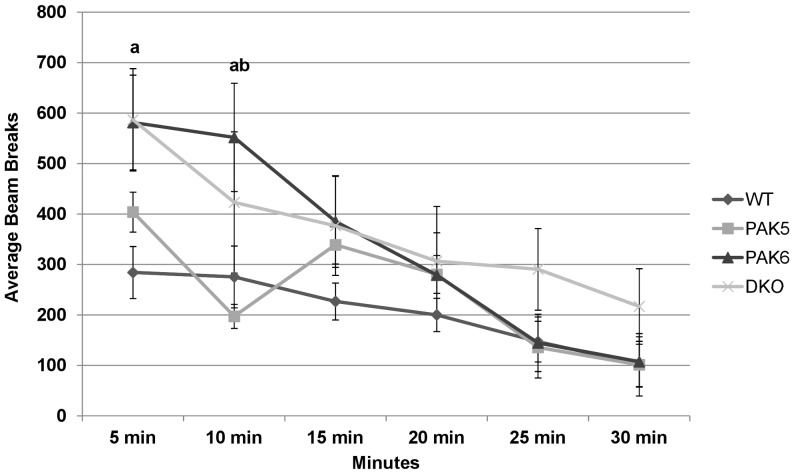
Third run- activity over 30 minutes. During the first 5 minute bin the PAK6 knockout mice were significantly more active compared to the wild type mice and during the second 5 minute bin, the PAK6 knockout mice were more active compared to the wild type mice and the PAK5 knockout mice. WT n = 8. PAK5 n = 5. PAK6 n = 8. DKO n = 5.

A repeated measures ANOVA of the third run of the open field activity assay revealed a significant effect of beam breaks per 5 minute bin [F(5, 100) = 29.080, p<0.0001] with activity significantly trending downward over the 30 minute testing period. There was also a significant interaction of genotype by bin [F(15, 100) = 2.816, p = 0.0011]. There was no significant difference in total number of beam breaks between genotypes. *Post hoc* tests for the first 5 minute bin showed that the wild type mice were significantly less active compared to the double knockout mice (p = 0.0130) and the *Pak6* knockout mice (p = 0.0082). *Post hoc* tests for the second five minute bin showed the *Pak6* knockout mice to be significantly more active than the *Pak5* knockout mice (p = 0.0154) and the wild type mice (p = 0.0349). *Post hoc* tests showed no significant differences between genotypes for the remaining 5 minute bins [Figure 4].

### All genotypes have normal anxiety levels

**Figure 5:**
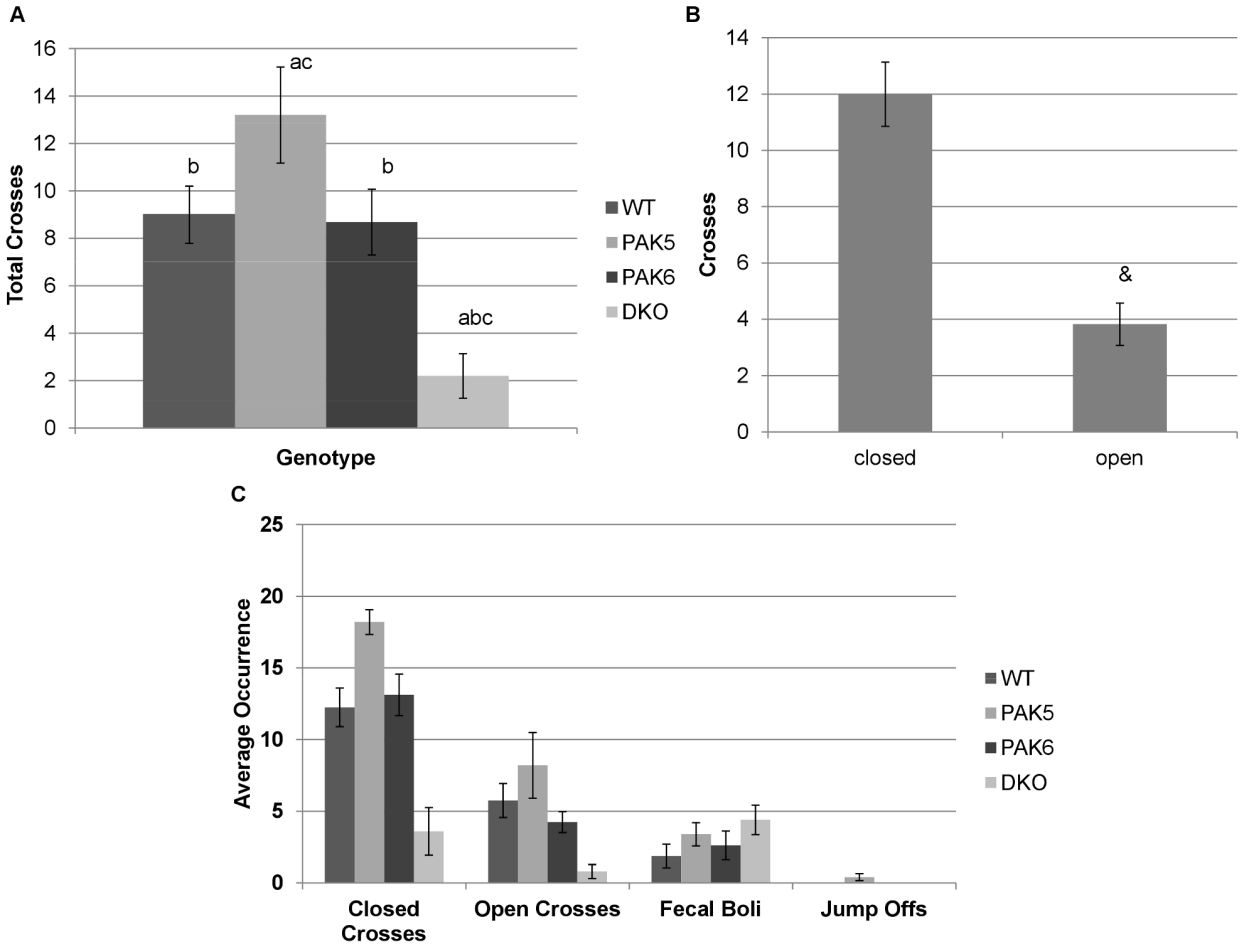
**a. Total Elevated Plus crosses**. The PAK5 knockout mice made more crosses into any arm of the elevated plus maze compared to the WT mice, the PAK6 knockout mice, and the DKO mice. The double knockout mice made significantly less crosses compared to the WT, PAK5 and PAK6 knockout mice. WT n = 8. PAK5 n = 5. PAK6 n = 8. DKO n = 5. **b. Open vs. Closed crosses.** The mice made significantly more crosses into the closed arms of the maze compared to the number of crosses they made into the open arms. (& = p<.05 compared to closed arms). WT n = 8. PAK5 n = 5. PAK6 n = 8. DKO n = 5. **c.** **Elevated plus maze.** There was no difference in number of fecal boli between the genotypes. Only the PAK5 knockout mice jumped off the apparatus. WT n = 8. PAK5 n = 5. PAK6 n = 8. DKO n = 5.

An ANOVA was used to analyze the data for the elevated plus maze. A significant difference in average total number of crosses was found between genotypes [F(3,44) = 17.991, p<0.0001] [Figure 5a]. A significant difference between type of cross (open arm or closed arm) was also found [F(1,44) = 52.997, p<0.0001] [Figure 5b]. There was no significant interaction between genotype and type of cross [F(3,44) = 2.349, p = 0.0855). *Post hoc* tests revealed that the *Pak5* knockout mice made significantly more crosses (p = 0.0037) and double knockout mice made significantly less crosses (p <0.0001) than the wild type mice. The *Pak6* knockout mice made significantly less crosses compared to the *Pak5* knockout mice (p = 0.0019) and significantly more crosses compared to the double knockout mice (p<0.0001). The *Pak5* knockout mice made significantly more crosses (p<0.0001) than the double knockout mice. The *Pak6* knockout mice and the wild type mice behaved similarly in number of crosses. There was no significant difference in number of fecal boli between the genotypes [F(3,22) = 1.221, p = 0.3663). There was an overall significant difference in number of jumpoffs [F(3,22) = 3.949, p = 0.0215) because the only genotype to have mice jump off the apparatus was the *Pak5* knockout mice [Figure 5c].

An ANOVA was run to analyze the percent of crosses into the open arm over the total number of crosses. No significant difference in the percent of open arm crosses was found between the genotypes.

### *Pak5* knockout mice and double knockout mice have deficits in rotorod performance

**Figure 6:**
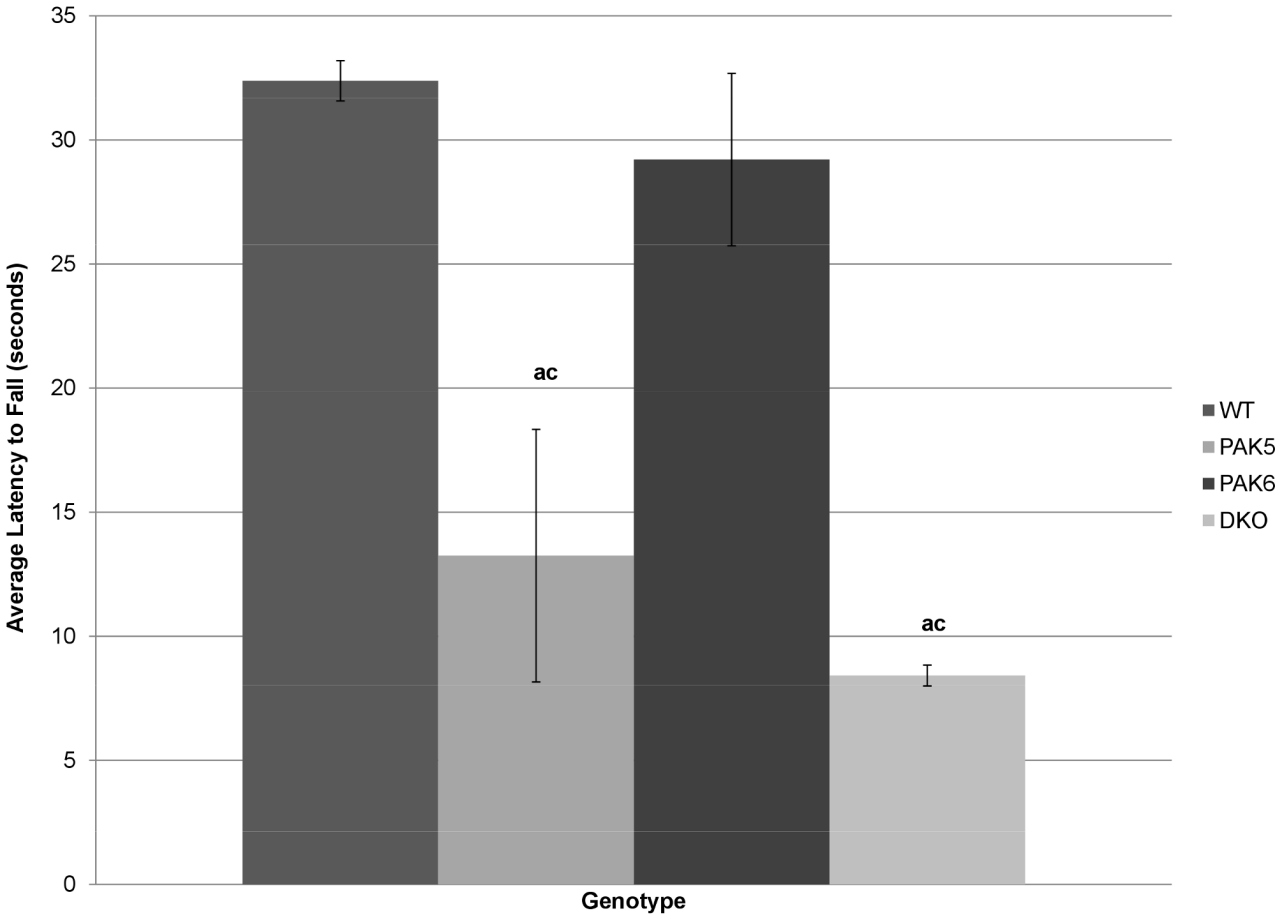
Latency to fall from the rotorod. The PAK5 knockout mice and the DKO mice fell from the rotorod faster than the wild type mice (a = p<.05 compared to WT) and the PAK6 mice (c = p<.05 compared to PAK6 knockout mice). WT n = 8. PAK5 n = 5. PAK6 n = 8. DKO n = 5.

The average latency to fall from the rotorod over three one minute trials was analyzed using an ANOVA. A significant difference between genotypes for latency to fall was found [F(3,8) = 14.263, p = 0.0014]. *Post hoc* tests revealed the *Pak5* knockout mice (p = 0.0025) and the double knockout mice (p = 0.0006) had significantly faster latency to fall from the rotorod when compared to the wild type mice. The *Pak5* knockout mice (p = .0067) and the double knockout mice (p = .0015) also had significantly faster latency to fall compared to the *Pak6* knockout mice. The *Pak5* knockout mice and the double knockout mice were not found to be significantly different from each other. The *Pak6* and the wild type mice were not found to be significantly different from each other [Figure 6].

### Double knockout mice may be less social than other genotypes

**Figure 7:**
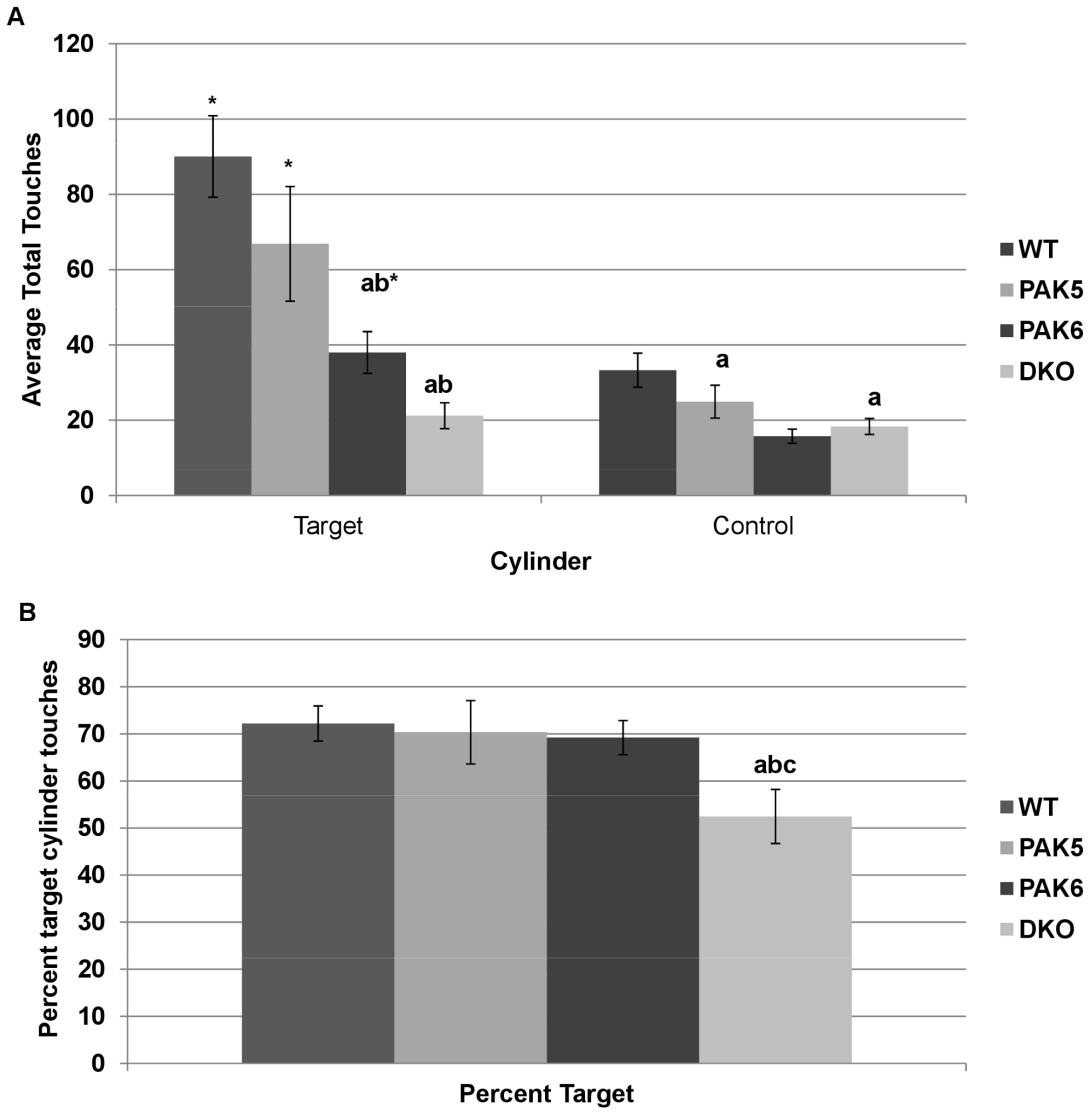
**a. Total touches to target and control cylinders**. The WT, PAK5 and PAK6 knockout mice made significantly more touches to the target cylinder compared to the control cylinder (* = p<.05 compared to control). The PAK6 knockout mice and the DKO mice made significantly less touches to the target cylinder compared to the number of touches the WT (a) and PAK5 knockout mice (b) made. The PAK6 knockout mice and the DKO mice made significantly less touches to the control cylinder compared to the WT mice. WT n = 8. PAK5 n = 5. PAK6 n = 8. DKO n = 5. **b. Percent touches to the target cylinder.** The double knockout mice had a significantly lower percentage of contact with the target cylinder compared to the WT, PAK5, and PAK6 knockout mice. WT n = 8. PAK5 n = 5. PAK6 n = 8. DKO n = 5.

A repeated measures ANOVA was used to analyze the results of the social chambers assay. Data points that exceeded 500 touches were excluded. A significant effect of genotype [F(3, 20) = 16.122, p<0.0001], a significant effect of which cylinder was touched [F(1, 20) = 31.867, p<0.0001], and a significant interaction of genotype by cylinder[F(3,20) = 4.609, p = 0.0131] was found. *Post hoc* tests further found the double knockout mice (p = 0.0076) and the *Pak6* knockout mice (p = 0.0011) touched the control cylinder significantly less times than the wild type mice. It was found that the *Pak6* knockout mice (p = 0.0005) and the double knockout mice (p<0.0001) touched the target cylinder significantly less times than the wild type mice. The *Pak6* knockout mice (p = 0.0492) and the double knockout mice (p = 0.0061) touched the target cylinder significantly less times than the *Pak5* knockout mice. The *Pak6* knockout mice (p = 0.0074), the *Pak5* knockout mice (p = 0.0481), and the wild type mice (p = 0.0061) touched the target cylinder significantly more times than the control cylinder. The double knockout mice touched both cylinders a similar number of times [Figure 7a].

An ANOVA was run to analyze the percentage of touches to the target cylinder over the total touches to any cylinder. There was an overall effect of genotype [F(3,20) = 3.293, p = 0.0417]. *Post hoc* tests revealed that the DKO mice had a significantly lower percentage of target cylinder touches compared to wild type mice (p = 0.0089), *Pak6* knockout mice (p = 0.0230), and *Pak5* knockout mice (p = 0.0245) [Figure 7b].

### Pak knockout mice are less aggressive than wild type mice

An ANOVA was run to analyze the average number of attacks initiated by the resident mice over the testing period. A significant difference in number of attacks between genotypes was found [F(3,19) = 3.622, p = 0.0320]. *Post hoc* tests showed that the wild type mice attacked significantly more times than the *Pak5* knockout mice (p = 0.0127), the *Pak6* knockout mice (p = 0.0476), and the double knockout mice (p = 0.0102).

**Figure 8:**
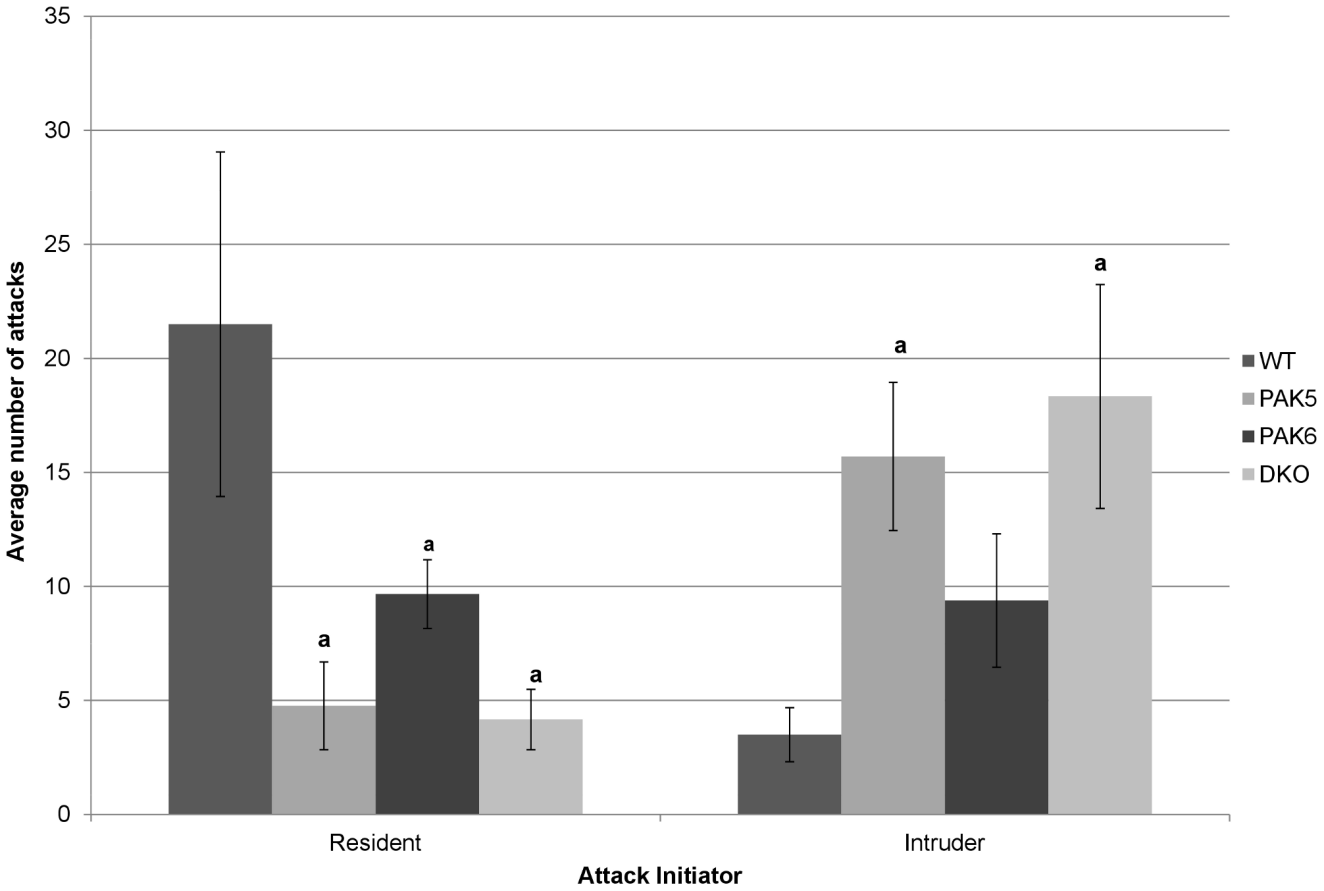
Resident/intruder paradigm. The PAK5, PAK6, and DKO mice initiated significantly less attacks on the intruder mice, compared with the WT mice. The PAK5 and DKO mice were attacked significantly more times by the intruder mice than the WT mice. WT n = 8. PAK5 n = 5. PAK6 n = 8. DKO n = 5.

An ANOVA was run to analyze the average number of attacks initiated by the intruder mice over the testing period. A significant difference in number of attacks committed between genotypes was found [F(3,19) = 4.253, p = 0.0185]. *Post hoc* tests revealed that the double knockout mice (p = 0.0044) and the *Pak5* knockout mice (p = 0.0156) were attacked significantly more times than the wild type mice [Figure 8].

### Double knockout mice have deficits in learning and memory

#### Acquisition Phase

**Figure 9:**
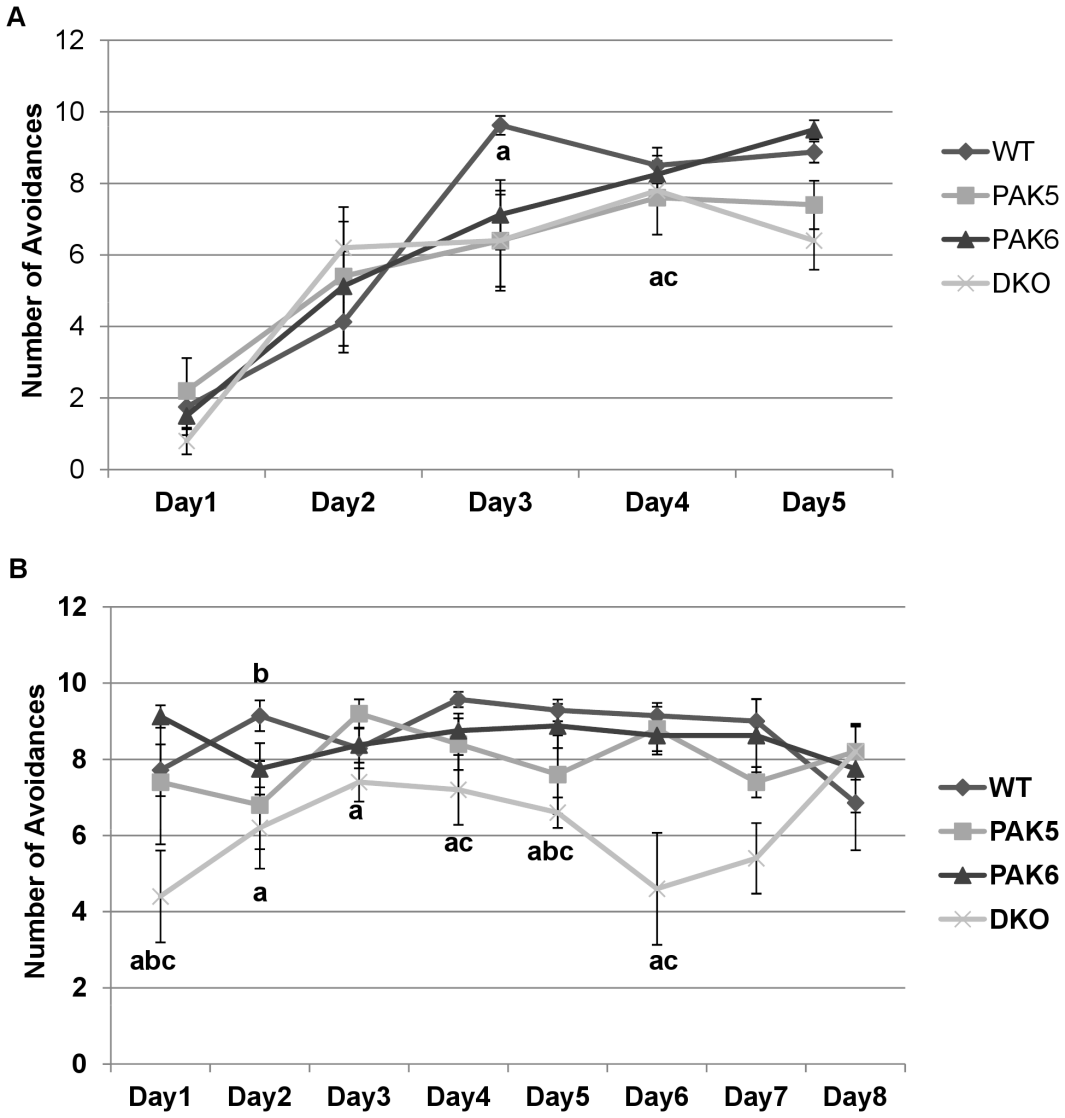
**a. Acquisition phase for active avoidance**. The DKO and PAK5 knockout mice made significantly less avoidances compared to the PAK6 and WT mice by the end of the 5 days of testing. WT n = 8. PAK5 n = 5. PAK6 n = 8. DKO n = 5. **b. Retention phase for active avoidance.** The DKO mice made less avoidances compared to the WT, PAK5, and PAK6 knockout mice over the 8 days of testing. WT n = 8. PAK5 n = 5. PAK6 n = 8. DKO n = 5.

A repeated measures ANOVA was run to analyze the average number of avoidances. No overall significance was found between genotypes [F(3,88) = .932, p = .4420]. An overall significant difference in the number of avoidances over the 5 days of testing was found [F(4, 88) = 58.104, p<0.0001]. The mice made more avoidances on days 2–5 then they did on day 1 (p<0.0001) and they made more avoidances on day 2 compared to day 1,3–5 (p<0.0001). On days 3, 4, and 5 there was no significant difference in the number of avoidances made. There was a genotype by day interaction [F(12,88) = 2.323, p = 0.0165]. On day 3 of testing the wild type mice made significantly more avoidances compared to the DKO mice (p = 0.0291) and compared to the *Pak5* knockout mice (p = 0.0291). On day 5 of testing, the wild type mice made more avoidances than the DKO mice (p = 0.0014) and the *Pak5* knockout mice (p = 0.0411). Also on day 5 of testing the *Pak6* made significantly more avoidances than the DKO mice (p = 0.0002) and the *Pak5* knockout mice (p = 0.0054) [Figure 9a].

#### Retention Phase

A repeated measures ANOVA was run to analyze the average number of avoidances made over the 8 days of testing. An overall significant difference between the genotypes was found [F(3,147) = 3.764, p = 0.0263]. *Post hoc* tests revealed the DKO mice made significantly less avoidances compared to the wild type mice (p = 0.006) and the *Pak6* knockout mice (p = 0.0076). An overall significant difference in number of avoidances was found over days of testing [F(7,147) = 2.121, p = 0.0448]. *Post hoc* test revealed that the number of avoidances made by all the mice on day 1 was significantly less than the number made on day 3(p = 0.0374), day 4 (p = 0.0064), and day 5(p = 0.0468). The number of avoidances made on day 2 was significantly less compared to day 4 (p = 0.0234). The number of avoidances made on day 4 was significantly more than the number on day 8 (p = 0.0297). A significant interaction of day by genotype was also found [F(21, 147) = 2.540, p = 0.0006]. *Post hoc* tests revealed that on day 1 the DKO mice made significantly less avoidances compared to the wild type mice (p = 0.0203), the *Pak5* knockout mice (p = 0.0476), and the *Pak6* knockout mice (P = 0.0014). On day 2 the DKO mice made significantly less avoidances than the wild type mice (p = 0.189). On day 3 the DKO mice made significantly less avoidances compared to the *Pak5* knockout mice (p = 0.0298). On day 4 the DKO mice made significantly less avoidances compared to the wild type mice (p = 0.0074). On day 5 the DKO mice made significantly less avoidances compared to the wild type mice (p = 0.0057) and compared to the *Pak6* knockout mice (p = 0.0141). On day 6 the DKO mice made significantly less avoidances than the wild type mice (p = 0.0003), the *Pak5* knockout mice (p = 0.0014), and the *Pak6* knockout mice (p = 0.0008). On day 7 the DKO mice avoided the shock significantly less times compared to the wild type mice (p = 0.0063) and the *Pak6* knockout mice (p = 0.0109) [Figure 9b].

### Summary

Table 1 presents an overview summary of these data.

**Table 1:** Summary of results and conclusions.

Experiment	Results/Conclusions
**Weight**	*Pak6* weigh more than other genotypes
**Open Field Activity**	In the 1^st^ run the DKO were less active than other genotypes. In the 2^nd^ run the DKO were less active than other genotypes. In the 3^rd^ run there were no differences.
**Elevated Plus Maze**	There was normal anxiety across groups; the mice made more crosses to closed than open. The *Pak5* made more total crosses than the WT. The DKO made less total crosses. The DKO results may be explained by motor deficits.
**Rotorod**	WT and *Pak6* performed similarly with normal motor coordination and balance. *Pak5* and DKO performed worse than WT and *Pak6*, with deficits in normal motor coordination and balance.
**Social Chambers**	WT, *Pak6*, and *Pak5* all made more contact with target. The DKO had lower percent contact with the target. The DKO difference may just be locomotor, but the percent contact does provide some evidence of less social behavior.
**Resident/Intruder**	All *Pak* knockout mice initiated less attacks to the intruder compared to WT mice. *Pak5* and DKO were attacked more by the intruder than the WT or *Pak6*. Pak knockouts are less aggressive, particularly P*ak5* and DKO.
**Active Avoidance**	In the acquisition phase, all mice learned to avoid the shock. On day 3 and 5, *Pak5* and DKO made less avoidances indicating subtle deficits in learning and memory. In the retention phase the WT, *Pak5* and *Pak6* avoided the shock. The DKO performed significantly worse. The DKO may have deficits in learning and memory.

WT = wild type mice, *Pak5*  =  *Pak5* knockout mice, *Pak6*  =  *Pak6* knockout mice. DKO =  *Pak5/Pak6* double knockout mice.

## Discussion

Our previous studies have shown the *Pak5/Pak6* double knockout mice to have higher body weights compared with wild type mice and to have deficits in learning and locomotion [18]. The present study assessed if the single knockout of either gene produced similar results. It was found that the *Pak6* knockout mice gained significantly more weight over the course of this study compared with the other three genotypes (wild type, *Pak5* knockout, and DKO). It was also found that, while at 5 months of age the *Pak6* knockout mice weighed similarly to the wild type mice and that both the *Pak6* knockout mice and the wild type mice weighed more than the *Pak5* knockout mice and the double knockout mice, at every time point after that the *Pak6* knockout mice weighed significantly more than all other genotypes. A possible reason for this weight gain could be related to the role that PAK6 has in inhibiting the activity of the androgen receptor. PAK6 lowers the level of transcriptional activity of both the androgen receptor and estrogen receptor [9], [13]. Androgen receptor knockout mice have been shown to have increased body fat [19]. It was suggested that the increased body fat resulting from the deletion of androgren receptor could be due to decreased lipolysis considering other findings that androgens are lipolytic [19].

Consistent with prior findings, the double knockout mice were shown to have decreased locomotor activity. During the first run of the open field activity assay, the double knockout mice were found to move significantly less than the other three genotypes. The same result was found in the second run of the open field activity assay. By the third run of this assay there was no significant difference in activity between any of the genotypes. Interestingly, the *Pak6* knockout mice and the *Pak5* knockout mice had similar activity levels as compared to the wild type mice. Therefore, there may be a functional redundancy of the *Pak5* and *Pak6* genes in motor activity and both genes need to be knocked out to display a deficit.

The elevated plus maze is an assay to model anxiety in rodents. Mice that enter the open arms of the maze are less anxious, while those that avoid the open arms and stay in the closed arms are more anxious. The number of fecal boli is also counted as a measure of anxiety, whereby more fecal boli would indicate a higher level of anxiety. No significant difference between genotypes for the percent of crosses into the open arm was found. There was no difference in the number of fecal boli between the genotypes either. All the genotypes therefore had similar levels of anxiety. The number of times the mice crossed from one arm to another was significant between genotypes. This however, appears to be another indication of the locomotor deficits of the double knockout mice as compared to the other genotypes, rather than a difference in anxiety level. Overall, all the genotypes entered the closed arm significantly more than the open arm of the maze.

The rotorod is a measure of motor function and coordination as well as balance. A significant difference was found between the genotypes in the latency to fall from the rotorod. The wild type mice and the *Pak6* knockout mice performed similarly and were able to remain on the rotorod longer than the *Pak5* knockout mice and the double knockout mice. The *Pak5* knockout mice and the double knockout mice performed similarly and poorly. This deficit in motor coordination could be attributed to the *Pak5* gene, because both the *Pak5* knockout mice and the double knockout mice performed poorly, while the *Pak6* knockout mice performed similarly to the wild type mice. These results are not consistent with the results of the open field activity assay. In the open field activity assay the *Pak5* knockout mice were found to have similar activity levels to the wild type mice. The open field activity assay only evaluates self-motivated horizontal movement. The rotorod test evaluates coordination and balance rather than just activity. Perhaps the deficits found in the *Pak5* knockout mice in the rotorod test can further focus future exploration into the motor neuron deficits seen in *Pak* knockout mice.

The social chambers assay revealed relatively normal social behavior of all the mice. The wild type mice, the *Pak5* knockout mice, and the *Pak6* knockout mice made contact with the target cylinder containing another mouse more times than they made contact with the cylinder containing nothing. This indicated that the wild type mice and single knockout mice had normal social behavior because they had a preference for the cylinder containing a mouse. Based on total contact with a cylinder it was unclear how social the double knockout mice were because they contacted both the target cylinder and the control cylinder less than the wild type mice. Therefore, the percent of contact with the target cylinder was evaluated to determine if the mice had a preference for the target cylinder. The percentage of target touches for the wild type, Pak5 knockout mice and Pak6 knockout mice were similar, while the percent of target cylinder touches for the double knockout mice was significantly lower the other genotypes. This indicates that the double knockout mice may be less social compared to the wild type mice and the *Pak5* knockout mice and the *Pak6* knockout mice.

The resident/intruder paradigm evaluates aggression in mice through the number of attacks a resident mouse initiates on an intruder mouse. The wild type mice were found to initiate significantly more attacks on an intruder than the *Pak5* knockout mice, the *Pak6* knockout mice or the double knockout mice. This is consistent with a previous finding that the double knockout mice were less aggressive than wild types in the resident/intruder paradigm [18]. This lack of aggression and lower social levels of the double knockout mice could correspond with their decreased activity levels.

In the first phase of the learning and memory assay, the active avoidance test, all of the genotypes made more avoidance responses on days 2–5 of testing compared to day 1 and more avoidance responses on day 3–5 than they did on day 2, indicating they all learned. However, on day 3 and day 5 the double knockout mice and the *Pak5* knockout mice made less avoidance responses than the wild type and therefore did not learn as well. In the retention phase, the double knockout mice once again performed significantly worse than the wild type and the *Pak6* knockout mice. This suggests that both the *Pak5* gene and the *Pak6* gene need to be knocked out for learning and memory impairments.

The deficits in locomotor activity in the double knockout mice are consistent with the results of the previous study [18]. It was also shown that the cortical neurons of these double knockout mice had fewer neurite outgrowths and abnormal filopodia formation [18]. Therefore, the malformation of the neurons and defects in the nervous system could lead to the functional deficits seen in the double knockout mice in our results. The double knockout mice had decreased size and improper location of growth cones in their cortical neurons which could explain the deficits in learning and memory [18]. These defects can result from changes in the cytoskeletal dynamics. It has been shown that the neuronal cytoskeleton regulates dendritic spine morphology and rearrangement of synaptic contacts, which are considered essential for learning and memory [20], [21]. In addition, PAK4 is important for motor neuron development indicating that the group B *Paks* are involved in motor function [17]. Collectively, our data indicate that, while these mice show no overt functional deficits, the *Pak5*, *Pak6*, and *Pak5/Pak6* knockout mice do exhibit subtle differences in body weight, locomotor activity, rotorod performance, and aggression.
